# Electrospun PANI/PEO-Luffa Cellulose/TiO_2_ Nanofibers: A Sustainable Biocomposite for Conductive Applications

**DOI:** 10.3390/polym17141989

**Published:** 2025-07-20

**Authors:** Gözde Konuk Ege, Merve Bahar Okuyucu, Özge Akay Sefer

**Affiliations:** 1 Mechatronics Program, Gedik Vocational School, Istanbul Gedik University, 34913 Istanbul, Türkiye; gozde.konuk@gedik.edu.tr; 2 Department of Mechatronics Engineering, Technology Faculty, Marmara University, 34722 Istanbul, Türkiye; merveokuyucu@marun.edu.tr

**Keywords:** luffa, titanium dioxide, electrospinning, nanofiber, biopolymer

## Abstract

Herein, electrospun nanofibers composed of polyaniline (PANI), polyethylene oxide (PEO), and *Luffa cylindrica* (LC) cellulose, reinforced with titanium dioxide (TiO_2_) nanoparticles, were synthesized via electrospinning to investigate the effect of TiO_2_ nanoparticles on PANI/PEO/LC nanocomposites and the effect of conductivity on nanofiber morphology. Cellulose extracted from luffa was added to the PANI/PEO copolymer solution, and two different ratios of TiO_2_ were mixed into the PANI/PEO/LC biocomposite. The morphological, vibrational, and thermal characteristics of biocomposites were systematically investigated using scanning electron microscopy (SEM), Fourier transform infrared spectroscopy (FTIR), X-ray diffraction (XRD), differential scanning calorimetry (DSC), and thermogravimetric analysis (TGA). As anticipated, the presence of TiO_2_ enhanced the electrical conductivity of biocomposites, while the addition of Luffa cellulose further improved the conductivity of the cellulose-based nanofibers. FTIR analysis confirmed chemical interactions between Luffa cellulose and PANI/PEO matrix, as evidenced by the broadening of the hydroxyl (OH) absorption band at 3500–3200 cm^−1^. Additionally, the emergence of characteristic peaks within the 400–1000 cm^−1^ range in the PANI/PEO/LC/TiO_2_ spectra signified Ti–O–Ti and Ti–O–C vibrations, confirming the incorporation of TiO_2_ into the biocomposite. SEM images of the biocomposites reveal that the thickness of nanofibers decreases by adding Luffa to PANI/PEO nanofibers because of the nanofibers branching. In addition, when blending TiO_2_ nanoparticles with the PANI/PEO/LC biocomposite, this increment continued and obtained thinner and smother nanofibers. Furthermore, the incorporation of cellulose slightly improved the crystallinity of the nanofibers, while TiO_2_ contributed to the enhanced crystallinity of the biocomposite according to the XRD and DCS results. Similarly, the TGA results supported the DSC results regarding the increasing thermal stability of the biocomposite nanofibers with TiO_2_ nanoparticles. These findings demonstrate the potential of PANI/PEO/LC/TiO_2_ nanofibers for advanced applications requiring conductive and structurally optimized biomaterials, e.g., for use in humidity or volatile organic compound (VOC) sensors, especially where flexibility and environmental sustainability are required.

## 1. Introduction

Environmental issues have compelled many scientists to investigate biodegradable, sustainable, non-toxic, and recyclable materials instead of petroleum-based materials, which are among the main causes of pollution on account of their non-biodegradability [1]. Among these materials, natural fibers are the most promising on account of their abundance, availability, and low cost, making them applicable in multiple industries, such as in medical applications [2], the aerospace [3] and automobile industries [4], water treatment [5], and food technology [6]. Natural fibers can have different physical and mechanical properties depending on whether they are plant-based (jute, flax, kenaf, Luffa, cotton, sisal) or animal-based (silk, wool, feathers, hear). Plant-based natural fibers are lignocellulosic fibers, which means they contain lignin, hemicellulose, and cellulose, and their composition may also change according to where they are grown. The *Luffa cylindrica* plant is a member of cucurbitaceous family, growing widely in the tropical regions of Asia, Africa, and south America [7]. Many studies have been conducted on *Luffa cylindrica* to examine its mechanical [8,9], chemical [10,11,12], and acoustic [13,14] properties due to its abundance, cheap price, non-toxicity, and good morphological structures, which enables its strong adherence to the matrix [15]. However, in order to enhance the adhesion between the fibers and the polymeric matrices in composites, it is necessary to subject the fibers to physical or chemical treatment [16,17]. These properties make it applicable in several domains, such as in pharmaceutical [18], biotechnology [19], and environmental contexts [20,21].

Nanomaterials, such as nanoparticles, nanorods, and nanofibers, exhibit a wide range of novel electrical, optical, mechanical, and chemical characteristics when compared to bulk materials [22,23]. This is why they find extensive use in sensor technologies, aerospace, defense, healthcare, automotive, and marine industries. Nanofibers have attracted significant attention due to their high surface-to-volume ratio, high porosity, excellent thermal stability, and precisely regulated diameter size. Electrospinning is a widely used method to fabricate nanofibers because of their adjustable morphological and mechanical properties, attained through control of the process conditions (electric voltage, the distance between syringe tip and collector, the feeding rate of syringe). Furthermore, obtaining homogeneous, continuous, and uniform fibers requires that we pay attention to the polymer characteristics, including viscosity, conductivity, surface tension, and molecular weight [24]. For instance, the diameter of the fibers can be reduced by conductivity, but viscosity can cause an increase in the diameter of fibers [25,26]. Polymers are always good candidates through which to obtain nanofibers via electrospinning; however, ceramic [27,28], metal oxides (TiO_2_ [29,30], ZnO [31]), and glass fiber [32] are also used in attaining nanocomposite fibers.

Natural fibers can be used as a hybrid biocomposite consisting of more than one kind of natural fiber or can be used to reinforce composites [33]. There are many polymers that are used as a blending polymer, such as polyethylene oxide (PEO), polyaniline (PANI), polyvinylpyrrolidone (PVP), polyvinyl alcohol (PVA), polythiophene (PT), and polypyrrole (PPY). Among them, conductive polymers are widely used because they offer a significant surface area, which enables them to exhibit better physical and chemical adsorption, as well as improved electrical properties. Additionally, they promote the generation of electron–hole pairs, hence enhancing the performance of electronic devices, strain sensor [34], gas sensors [35,36], organic photovoltaic (OPV) devices [37], and energy storage [38]. PANI’s high conductivity, easy synthesis, low cost, and strong stability have brought it to the forefront of many studies in the literature [38].

In recent years, conducting polymer–metal oxides [39,40], conducting polymer–natural fibers [41,42], and polymer–natural fibers–metal oxide nanocomposites have received great interest in industrial applications, including electronic, biomedical, and batteries technologies, owing to their improvement of important polymer properties. Bharatraj Singh Rathore et al. have synthesized a biocomposite using chitosan, polyaniline, and nickel oxide [43]. Polymeric nanocomposites consisting of polyaniline (PANI)/tin dioxide (SnO_2_) and PANI/chitosan (CS)/SnO_2_ were chemically produced using the in situ polymerization process by A.L.C. Silva et al. [44]. Yongqiang Shi et al. have fabricated a microfiber sensor the evaluated the freshness of pork via a titanium dioxide-polyaniline/silk fibroin fiber (TiO_2_-PANI/SFF biocomposite [45].

In the present study, a conductive electrospun Luffa cellulose (LC)-based PANI/PEO/LC with TiO_2_ nanoparticles was synthesized using the electrospinning method. In our earlier research, PANI/PEO/Luffa-conductive biocomposite nanofibers were fabricated, and the effect of Luffa cellulose on PANI/PEO-conductive copolymer was discussed by comparing the PANI/PEO/LC electrospun membrane with different ratios of Luffa cellulose [46]. As stated before, PEO was used as a carrier polymer for the electrospinning process. The main focus of this study is to investigate the effect of TiO_2_ nanoparticles on membrane morphology, including the orientation and diameter of nanofibers in the PANI/PEO/LC/TiO_2_ biocomposite nanofiber membrane. Hence, the novelty of the present work lies in its offering an original relation between natural fibers (LC), conductive polymers (PANI), and metal oxide nanoparticles (TiO_2_) as a hybrid system. For this purpose, titanium dioxide (TiO_2_) was added to the Luffa-based PANI/PEO copolymer at two different mass ratios. First, alkali treatment was carried on *Luffa cylindrica* to remove hemicellulose, lignin, and some waxy materials. Then, PANI, PEO, Luffa cellulose, and TiO_2_ were blended, and the electrospun PANI/PEO, PANI/PEO/LC, PANI/PEO/LC/TiO_2_, and PANI/PEO/LC/TiO_2_:2 nanofiber mats were fabricated via the electrospinning method. The conductivity and viscosity of biopolymer solutions were measured using a conductivity meter and viscometer. The yielding biocomposite nanofibers were characterized using Fourier transform infrared spectroscopy (FTIR), scanning electron microscope (SEM), X-ray diffraction (XRD), differential scanning calorimetry (DSC), and thermogravimetric analysis (TGA).

## 2. Materials and Methods

### 2.1. Materials

The *Luffa cylindrica* utilized in this work originate from Turkey. PANI (polyaniline), with a molecular weight of 50,000; PEO (polyethylene oxide), with a molecular weight of 900,000, CSA (camphor sulfonic acid); SDS (sodium dodecyl sulfate); and titanium dioxide (TiO_2_) were acquired from Sigma-Aldrich (Steinheim, Germany). The chloroform and dimethylformamide (DMF) used to create the PANI/PEO solution, as well as ethanol, xylene, sodium hydroxide (NaOH), hydrochloric acid (HCl), and other chemicals, were obtained from Merck Company (Darmstadt, Germany). Other commercial suppliers supplied guaranteed-grade reagents. These chemicals were utilized without any additional purification.

### 2.2. Alkali Treatment of Luffa Fibers and Preparation of Biocomposites

**Figure 1:**
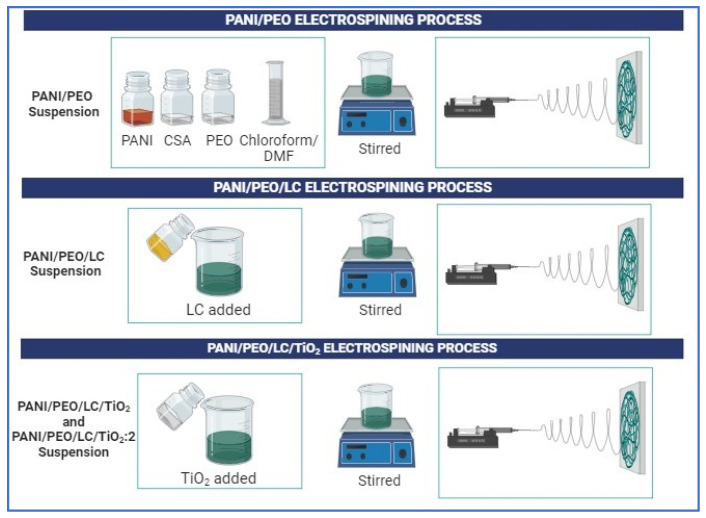
Schematic representation of fabrication of electrospun PANI/PEO, PANI/LC, PANI/PEO/LC/TiO_2_, and PANI/PEO/LC/TiO_2_:2 composites.

The electrospinning suspension comprised cellulose from Luffa, PANI, PEO, and TiO_2_, with four distinct samples prepared, namely, PANI/PEO, PANI/PEO/LC, PANI/PEO/LC/TiO_2_, and PANI/PEO/LC/TiO_2_:2, as illustrated in Figure 1. First, alkali treatment was carried on *Luffa cylindrica* to remove hemicellulose, lignin, and some waxy materials from it. Then, PANI, PEO, Luffa cellulose, and TiO_2_ were blended and the electrospun. PANI/PEO, PANI/PEO/LC, PANI/PEO/LC/TiO_2_, and PANI/PEO/LC/TiO_2_:2 nanofiber mats were fabricated via the electrospinning method. Cellulose extraction from Luffa was achieved through an alkaline treatment process. The alkali treatment process was detailed extensively in our previous study [46]. *Luffa cylindrica* initially develops as a fruit encapsulated by a green outer peel. Upon completion of the maturation process, the fruit undergoes natural drying. Subsequently, the dried Luffa fruit is mechanically processed using a grinder to obtain fibrous particulate matter suitable for further applications. PANI is used in its emeraldine base form, which exhibits insulating properties. To convert the polymer into its emeraldine salt form (its conductive form), doping is required. To achieve this transformation, camphor sulfonic acid (CSA) is employed as a doping agent, enabling the transition of polyaniline to its conductive state. In the initial step of the suspension preparation process, where all suspensions were prepared using the same component ratios, 2% *w*/*v* of PANI and 3% *w*/*v* of CSA (1:1.5 *w*/*w* ratio) were dispersed in a solvent mixture of chloroform and DMF at a 1:1 volume ratio (*v*/*v*) and stirred for 24 h at room temperature.

Subsequently, a solution containing 2% *w*/*v* of PEO was added as a carrier polymer and stirred for 24 h. Finally, a solution containing 0.5% *w*/*v* of SDS was introduced and stirred for 24 h. In the following step, TiO_2_ powder was added into the suspension in two different ratio (2% and 4%); suspensions were then stirred for 24 h. All composition ratios of PANI/PEO, PANI/PEO/LC, PANI/PEO/LCT/iO_2_, and PANI/PEO/LC/TiO_2_:2 are given in Table 1.

### 2.3. Electrospinning for Nanofiber Formation

The electrospinning process was carried out using the NE300 Nano Spinner device (Inovenso, Türkiye). An 18-gauge nozzle was used for the process. The polymer solutions were poured into a 10 mL syringe and placed onto the syringe pump. The applied voltage and collector rotation speed were optimized in our previous study [46]. In the present study, to determine the optimum fiber structure, the voltage and collector rotation speed were kept constant, while the distance between the needle tip and the collector, as well as the feeding rate, were selected as variable parameters. The electrospinning process was performed at a collector rotation speed of 300 rpm and an applied voltage of 30 kV. However, the addition of TiO_2_ increased the electrical conductivity of the solution. As the conductivity increases, the surface charge density also increases, leading to faster jet formation during the electrospinning process. This condition may result in instability in the fiber formation and the occurrence of bead defects. Furthermore, TiO_2_ also reduced the viscosity of the polymer solution. Considering these factors, the applied voltage was decreased to improve fiber morphology and to obtain more uniform, bead-free nanofibers. Production was carried out for 45 min. All data used in the process are presented in Table 2.

### 2.4. Instrumentations

To measure the viscosity and conductivity of composite solutions, the Brookfield viscometer and the Endress + Hauser conductivity meter were used, respectively. For viscosity measurements, the device’s spindle is submerged in the solvent and operated at a rotational speed of 100 revolutions per minute in the viscometer. To determine the vibrational properties of nanofiber membranes, the Perkin Elmer Spectrum 100 series FT-IR spectrometer was used. Each sample was subjected to 100 scans, with scan numbers ranging from 4000 to 650 cm^−1^, at a scan rate of 4.0 cm^−1^. X-ray diffraction (XRD) was performed with Bruker D8 Advance. All infrared spectra were taken at room temperature with a 2θ (5–40°), operated at 40 mA and 40 kV. An SEM (Zeiss-Evo|MA10) analysis was carried out to investigate the morphological properties of the yielded samples. The diameters of the electrospun nanofibers were measured using imageJ software at different points of the SEM images. The thermal characteristics were examined using differential scanning calorimetry (DSC). The Perkin Elmer Jade DSC instrument was utilized to treat the nanofiber copolymers thermally. Each cycle was completed under a nitrogen environment with a heating rate of 10 °C/min. TGA was performed using Mettler Toledo STARe, with a heating speed of 10 °C/min. The specimens were heated from room temperature to 600 °C in an argon environment.

## 3. Results and Discussion

In order to identify the electrospinning conditions (flow rate, applied voltage, and the distance between syringe tip and collector) and compare the solutions’ properties, the viscosity and conductivity of the biopolymer solutions were measured and are given in Table 1. Viscosity is an important feature that directly affects the morphology of nanofibers. As shown in Table 1, while cellulose increases the viscosity of the PANI/PEO copolymer solution, TiO_2_ considerably decreases the viscosity of the PANI/PEO/LC solution. The reason for the decreasing viscosity may be the changing molecular structure of the polymers, such as the molecular weight or the polymer chain [47]. The conductivity values of the copolymer PANI/PEO and the biopolymers PANI/PEO/LC, PANI/PEO/LC/TiO_2_, and PANI/PEO/LC/TiO_2_:2 are given in Table 1. According to results, the highest conductivity belongs to PANI/PEO/LC/TiO_2_:2 due to its containing a high quantity of TiO_2_. As mentioned before [46], Luffa cellulose elevates the conductivity of copolymer solutions due to its plasticizing effect.

Regarding the electrospinning process, Table 2 gives the optimized conditions for the desired nanofiber membranes for each copolymer solution. For each step, the collector speed has been kept fixed. According to the values obtained, after decreasing the viscosity, the smaller voltage led to finer nanofibers in the PANI/PEO/LC/TiO_2_ and PANI/PEO/LC/TiO_2_:2 processes.

### 3.1. FT-IR Spectra

**Figure 2:**
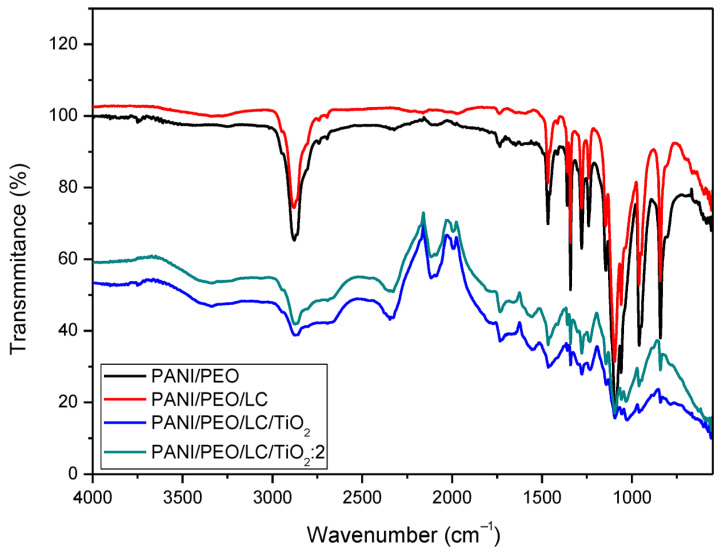
FT-IR spectra of PANI/PEO, PANI/PEO/LC, PANI/PEO/LC/TiO_2_, and PANI/PEO/LC/TiO_2_:2 composites nanofibers.

Figure 2 displays the FT-IR spectra of several materials. The spectra display the percentage of light transmitted by materials at specific wavelengths. By analyzing this data, one can acquire knowledge regarding the constituents and molecular connections of the materials. Specifically, the peaks observed in the 1580–1600 cm^−1^ and 1480–1500 cm^−1^ regions correspond to the quinonoid and benzenoid rings of PANI, while the peaks at 2870–2880 cm^−1^, 1450 cm^−1^, and 1100 cm^−1^ correspond to the asymmetric C-H stretching, CH2 bending, and C-O-C stretching vibrations of PEO [46,48]. In the PANI/PEO/LC FT-IR spectrum, the broad OH stretching vibration band observed in the 3500–3200 cm^−1^ range signifies the presence of hydroxyl (OH) groups [49]. The bands observed at the 3500–3200 cm^−1^ and 1200–900 cm^−1^ regions are indicative of the presence of cellulose. Given that cellulose contains a substantial amount of OH groups, the band in this region is more pronounced and broader in comparison to the PANI spectrum, which can be attributed to the addition of cellulose [50]. Additionally, the 1200–900 cm^−1^ region encompasses the characteristic C-O-C and C-OH vibrations of cellulose [51,52]. These bonds, inherent to the structure of cellulose, result in distinctive bands within this region. The increased intensity and number of bands in this region in the PANI/LC spectrum further confirm the presence of cellulose and suggest that the incorporation of cellulose into PANI significantly alters the chemical properties of the material. In the region between the peaks corresponding to Ti-O-Ti and Ti-O-C, vibrations attract attention [53,54]. These peaks confirm the presence of TiO_2_ and indicate the influence of TiO_2_ in the PANI/PEO/LC/TiO_2_ and PANI/PEO/LC/TiO_2_:2 composites.

### 3.2. XRD Analysis

**Figure 3:**
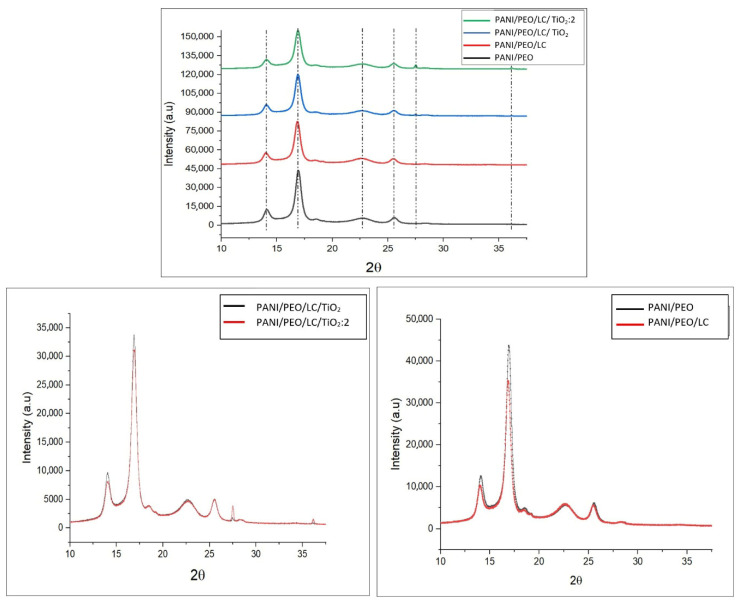
XRD spectra of PANI/PEO, PANI/LC, PANI/PEO/LC/TiO_2_, and PANI/PEO/LC/TiO_2_:2 composites nanofibers.

Figure 3 presents the X-ray diffraction (XRD) patterns of PANI/PEO, PANI/PEO/LC, PANI/PEO/LC/TiO_2_, and PANI/PEO/LC/TiO_2_(2X) within the 5–40° (2θ) range. The XRD pattern of PANI exhibits diffraction peaks at 14.104°, 16.940°, and 18.54°, with the peak at 16.94° being particularly indicative of the characteristic crystallinity of PANI. The doping of PANI with CSA (camphor sulfonic acid) has been observed to increase the intensity of this diffraction peak, while the peak at 14.104° is likely to have emerged due to CSA’s structural influence on PANI, as CSA enhances the regularity and crystallinity of the polymer. The amorphous nature of PEO (polyethylene oxide) contributes to a broad peak formation in the 15–19° range; however, the presence of CSA mitigates the amorphous effect of PEO, stabilizing the crystalline phase in specific regions. In the PANI/PEO/LC sample, the regenerated cellulose used belongs to the cellulose II crystalline form, which has been observed to largely preserve the crystalline phases of PANI. While the XRD peaks of PANI and PANI/PEO/LC are located in similar regions, the incorporation of LC enhances crystallinity in the 18–22° range, with the 28.279° peak becoming more pronounced, indicating the formation of a new crystalline phase due to PANI–LC interactions. The literature also indicates that cellulose reinforcement stabilizes the crystalline structure of the PANI matrix [55]. The XRD pattern of PANI/PEO/LC/TiO_2_ reveals diffraction peaks at 27.48°, 34.33°, 36.09°, and 54.35°, corresponding to the anatase phase of TiO_2_. In the PANI/PEO/LC/TiO_2_:2 sample, the increase in TiO_2_ concentration has led to the emergence of new crystalline phases, indicating an enhancement in the material’s crystallinity. However, the interaction between PANI and TiO_2_ results in weakened peak intensities [56,57,58,59]. These findings demonstrate that an increase in TiO_2_ concentration contributes to enhanced crystallinity in PANI-based systems, leading to a more structured and highly crystalline material.

### 3.3. SEM Images

**Figure 4:**
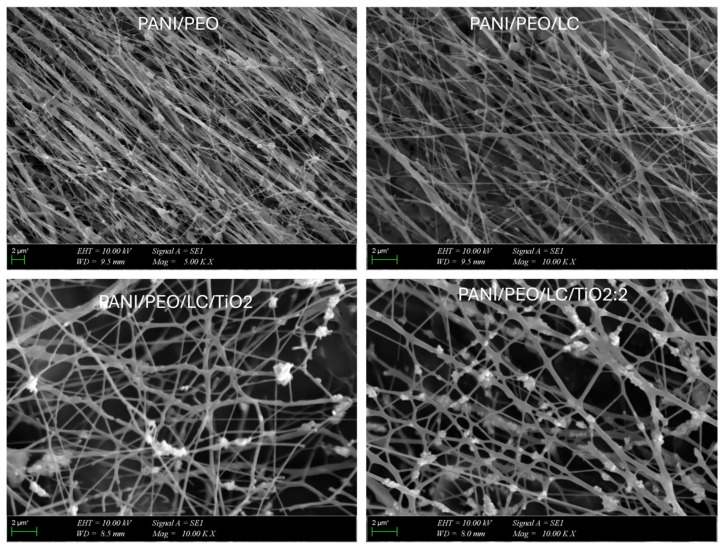
SEM micrographs of PANI/PEO, PANI/PEO/LC, PANI/PEO/LC/TiO_2_, and PANI/PEO/LC/TiO_2_:2 composites nanofibers.

The SEM analyses were used to investigate the morphology and diameter of composite nanofibers. As shown in Figure 4, the morphology and diameter of nanofibers were changed according to composition of composites. This variation could result from the changing rheology of the composites, which depends on the viscosity, concentration, and interfacial properties (electron transfer, electric double layer, surface charge transfer resistance) of the polymer solution [60]. According to the SEM results shown in Figure 4, cellulose extracted from *Luffa cylindrica* are added to the blended polymers increases the branching on the electrospun composite and decreases the fiber diameter since Luffa increases the viscosity of polymer solutions. Furthermore, cellulose also makes random fiber connections, which increase the porosity of the electrospun biocomposite when compared to the PANI/PEO copolymer nanofiber. This improvement in the porosity can provide better adhesion to surface membranes for the VOC sensor application, as mentioned above. When the amount of TiO_2_ in the blended polymer solution increases, the conductivity of the solution increases, which brings about a decrease in the fiber diameter of the electrospun copolymer. As reported by Khan et al., increasing the TiO_2_ concentration in the composite decreases the average diameter of the PVA/TiO_2_ nanocomposite [61]. Another important reason for obtaining thinner fibers is that the viscosity of the polymer solution decreases with the addition of TiO_2_, which brings about a decrease in the entanglement of, and interaction between, polymer chains [62]. Therefore, as a result of these membranes with thinner fibers increasing the volume surface ratio, there is a more porous structure and enhanced surface sensitivity, especially for sensor applications. Additionally, increasing the conductivity of the biocomposite solution leads to an increase in the surface charge of the polymer jet, which increases the elongation force, resulting in the formation of smoother and thinner fibers [57].

**Figure 5:**
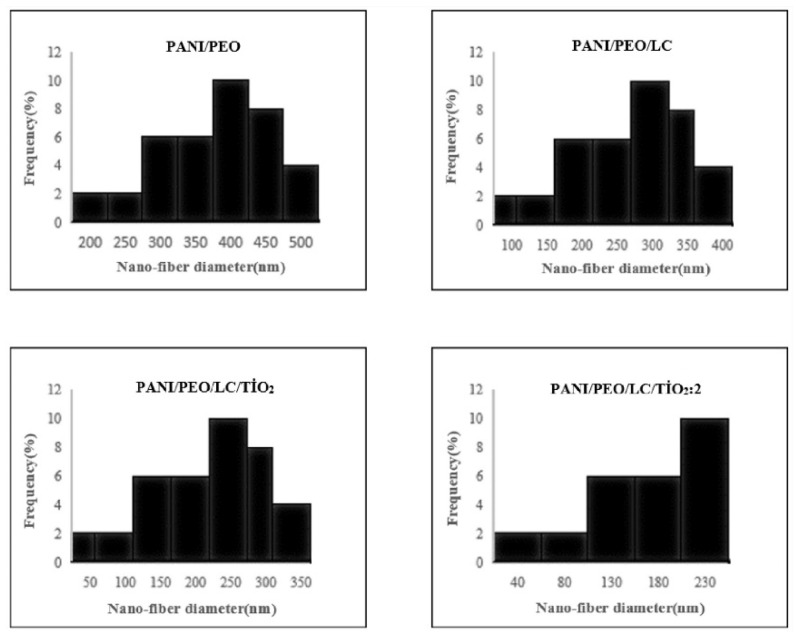
Diameter distribution of fibers of electrospun PANI/PEO, PANI/PEO/LC, PANI/PEO/LC/TiO_2_, and PANI/PEO/LC/TiO_2_:2 composites.

The diameter distributions of the biocomposite nanofibers are illustrated in Figure 5. The PANI/PEO nanofibers had a minimum diameter of 212 nm and a maximum diameter of 490 nm. Incorporating LC into the PANI/PEO nanocomposites resulted in more aligned, smaller, and uniform fibers, with diameters ranging from 134 nm to 389 nm. The PANI/PEO/LC/TiO_2_ nanocomposites exhibited even smaller diameters, ranging from 56 nm to 236 nm. The PANI/PEO/LC/TiO_2_:2 nanocomposites showed the smallest diameters, with a minimum of 42 nm and a maximum of 218 nm. The decrease in nanofiber diameter and increased branching are attributed to the enhanced solution conductivity. Higher solution conductivity facilitates the production of finer fibers and increases the droplet charge that forms the Taylor cone. The PANI/PEO/LC/TiO_2_:2 nanocomposite demonstrated the most optimized fiber distribution and diameter among all the samples.

### 3.4. DSC Analyses

**Figure 6:**
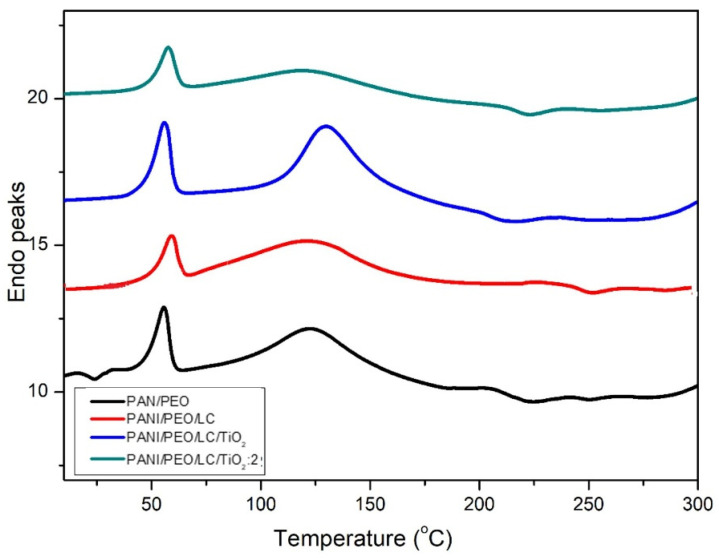
DSC curves of electrospun PANI/PEO, PANI/PEO/LC, PANI/PEO/LC/TiO_2_, and PANI/PEO/LC/TiO_2_:2 composites.

Differential scanning calorimetry (DSC) helps determine how the materials’ physical properties vary during the heating and cooling processes. Figure 6 shows the thermal behaviors of all specimens analyzed by DSC. Shifting peaks to higher temperatures, Luffa cellulose makes PANI/PEO copolymer nanofibers more brittle and decreases the free volume in the PANI/PEO/LC nanofibers. Regarding the glass transition temperature (Tg) of the samples, given in Table 3, the Tg values increased from 154.07 °C to 161.35 °C, and adding LC to the PANI/PEO electrospun composite means weakening the flexibility of the PANI/PEO membrane. Likewise, TiO_2_ improves the structural order of biopolymer nanofibers; the Tg value of PANI/PEO/LC/TiO_2_:2 composites increased to about 173.18 °C when titanium dioxide was added to the biopolymer nanofibers. Therefore, the crystallinity of biopolymers increases with TiO_2_, as reported in the XRD results of Fei et al. [63], and this increase is also an indication that the intermolecular force between the polymer chains increases and makes it more thermally stable. Hence, TiO_2_ significantly enhances thermal stability, making the composite more resistant to thermal degradation as also obtained from TGA thermograms. This improved thermal resistance can be beneficial for sensor applications, especially in environments with temperature fluctuations. According to Figure 6, all thermogram specimens show nearly the same behavior with respect to heat. The first peak around 50–65 °C occurs due to the evaporation of water from the composite nanofibers. The exothermic peak at 225 °C indicates the decomposition of composites for all specimens.

### 3.5. TGA Analysis

**Figure 7:**
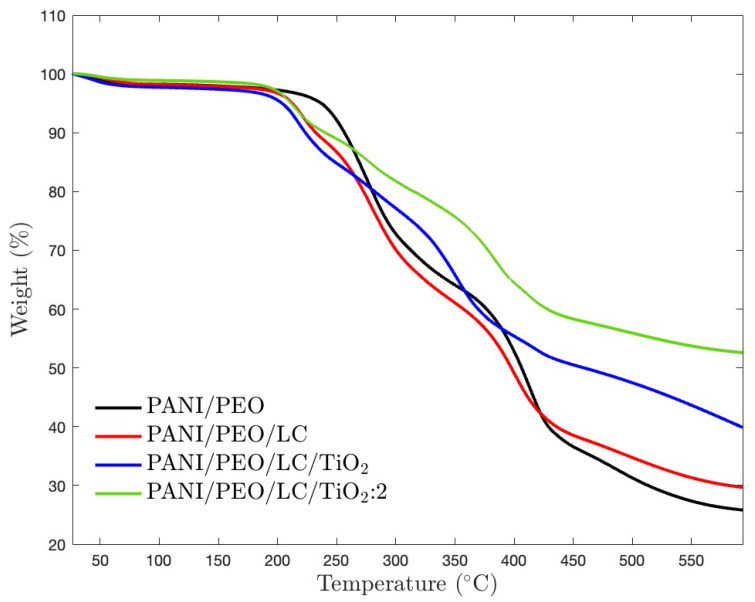
TGA thermograms of electrospun PANI/PEO, PANI/PEO/LC, PANI/PEO/LC/TiO_2_, and PANI/PEO/LC/TiO_2_:2 composites.

Thermogravimetric analysis (TGA) is used to investigate the thermal stability of materials that are crucial parameters for high-temperature applications. Figure 7 illustrates the thermogravimetric behavior of pure PANI/PEO and its composites with Luffa cellulose (LC) and TiO_2_. PANI/PEO has the lowest thermal stability due to the maximum mass loss (about 75%), as shown in Table 3. Incorporating LC into the PANI/PEO matrix slightly improves thermal stability compared to the pristine polymer blend. TiO_2_ nanoparticles increase the resistance to decomposition, which means higher thermal stability is also consistent with the XRD results that mention that TiO_2_ improves the crystallinity of the biocomposites, and DSC results state that TiO_2_ elevates the thermal stability of biocomposites. Therefore, PANI/PEO/LC/TiO_2_:2, containing more TiO_2_ nanoparticles, had minimal mass loss (about 50%). Hence, as also given in Table 3, LC and TiO_2_ improve the thermal stability of pristine PANI/PEO electrospun composites. Moreover, each sample follows three main degradation stages. Below 100 °C, the minor mass loss occurred due to the evaporation of absorbed moisture and residual solvents [64]. The second weight loss occurred between 180 and 420 °C as a result of the decomposition of the PEO chains and the degradation of hemicellulose and because of the backbone degradation of PANI and the complete decomposition of lignin and other organic components, which led to a weight loss of about 420 °C [65]. Furthermore, TiO_2_ led to upward shifting in the second degradation temperature from 229–415 °C to 240–446 °C, indicating increasing thermal stability due to more oriented nanofibers, resulting in more alignment polymer chains, as also supported by SEM results.

## 4. Conclusions

Hearin, a novel conductive biocomposite nanofiber based on polyaniline (PANI), polyethylene oxides (PEO), and Luffa cellulose (LC) with titanium dioxide nanoparticles (TiO_2_) was fabricated through electrospinning. The higher conductivity value was obtained from the PANI/PEO/LC/TiO_2_:2 biocomposite nanofiber. The FT-IR results verify the chemical formation between PANI/PEO and Luffa, broadening the peak in region 3500–3200 cm^−1^ the, and the new vibrations in the 400–1000 cm^−1^ region indicate the TiO_2_ nanoparticles on the biocomposite’s nanofiber membranes. Moreover, SEM images were quite compatible with the conductivity results, indicating that the PANI/PEO/LC/TiO_2_:2 membrane has the finest and thinnest fibers, and thicker fiber diameters were obtained on the PANI/PEO copolymer. Furthermore, the results obtained from X-ray diffraction (XRD) and differential scanning calorimetry (DSC) analyses suggest that the incorporation of cellulose into the nanofiber matrix adversely affected its flexibility, which is likely due to the rigid and crystalline nature of cellulose. In addition, the presence of titanium dioxide (TiO_2_) nanoparticles was found to enhance the overall crystallinity of the biocomposite nanofibers, which may contribute to improved structural integrity and thermal stability, as supported by TGA results. The most thermally stable composite is the one with the highest TiO_2_ content (PANI/PEO/LC/TiO_2_:2), suggesting its potential in high-temperature applications. These findings support the use of biopolymer and inorganic filler hybridization to improve the thermal performance of conductive polymer composites.

## Figures and Tables

**Table 1:** Viscosity and conductivity measurements and ratios of biocomposite suspensions.

Samples	PANI (% *w*/*v*)	PEO (% *w*/*v*)	CSA (% *w*/*v*)	LC (% *w*/*v*)	TiO_2_ (% *w*/*v*)	Viscosity (cp)	Conductivity (μS/cm)
PANI/PEO	2	3	3	-	-	412	446/24 °C
PANI/PEO/LC	2	3	3	2.5		487	473/24 °C
PANI/PEO/LC/TiO_2_	2	3	3	2.5	2	270	490/24 °C
PANI/PEO/LC/TiO_2_:2	2	3	3	2.5	4	293	531/24 °C

**Table 2:** Optimal electrospinning conditions of electrospun PANI/PEO, PANI/PEO/LC, PANI/PEO/LC/TiO_2_, and PANI/PEO/LC/TiO_2_:2 composites.

Sample	Distance Between Collector and Nozzle (cm)	Voltage (kV)	Flow Rate (mL/h)	Collector Speed (rpm)	Operation Time (min)
PANI/PEO	20	30	1.1	300	45
PANI/PEO/LC	20	30	1.1	300	45
PANI/PEO/LC/TiO_2_	16	23	1.0	300	45
PANI/PEO/LC/TiO_2_:2	15	23	1.4	300	45

**Table 3:** The glass transition (T_g_) and weight loss percentage values of electrospun PANI/PEO/LC, PANI/PEO/LC/TiO_2_, and PANI/PEO/LC/TiO_2_:2 composites.

	T_g_^a^ (°C)	Weight Loss ^b^ (%)
PANI/PEO	154.07	75
PANI/PEO/LC	161.35	70
PANI/PEO/LC/TiO_2_	168.93	60
PANI/PEO/LC/TiO_2_:2	173.97	50

^a^ Determined by DSC measurement. ^b^ Determined by TGA measurement.

## Data Availability

All data for the manuscript are available upon request.
